# P-1075. Treatment Patterns among Hospitalized Patients with Gram-Negative Infection Treated with Imipenem/Cilastatin/Relebactam (I/R): Retrospective US-based EHR Medical Chart Review

**DOI:** 10.1093/ofid/ofae631.1263

**Published:** 2025-01-29

**Authors:** Emre Yucel, Lulu Lee, Kushal Modi, Yanbing Zhou, Ryan K Shields

**Affiliations:** Merck & Co., Inc., North Wales, Pennsylvania; Oracle Life Sciences, Los Angeles, California; Oracle Life Sciences, Los Angeles, California; Merck, Rahway, New Jersey; University of Pittsburgh, Pittsburgh, Pennsylvania

## Abstract

**Background:**

Real world evidence with I/R is limited. This study describes the patient, treatment, and clinical outcomes of hospitalized adult patients with a suspected gram negative (GN) infection who were treated with I/R for ≤48hrs.

**Table 1.**
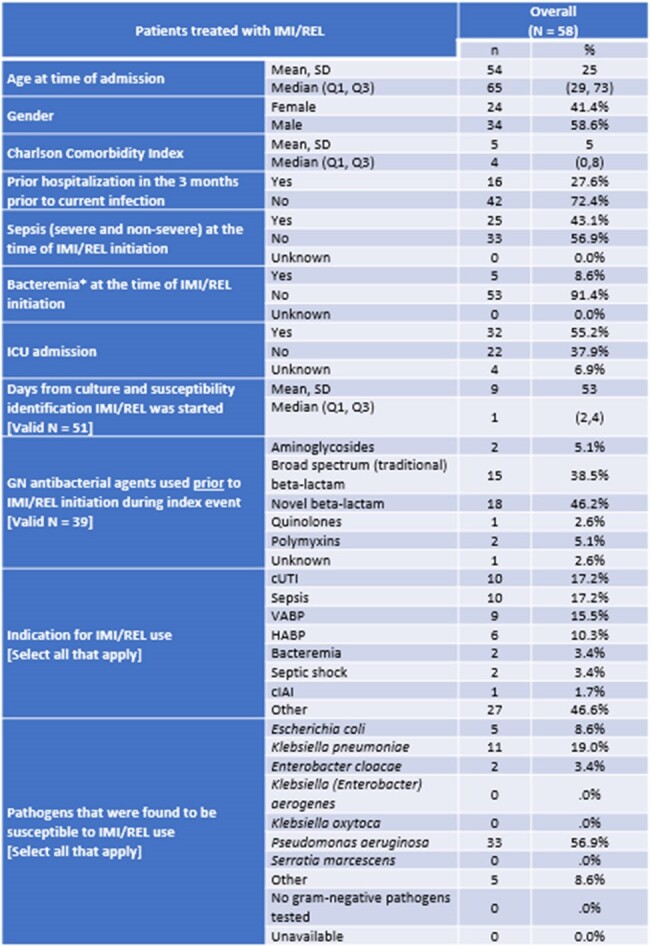
Initial baseline characteristics at index period for overall IMI/REL patients and stratified by COVID-19 at time of index SD, standard deviation.

**Methods:**

This retrospective manual electronic health records (EHR) chart review used Oracle Life Sciences’ EHR database, a large US health systems database capturing inpatient and outpatient encounters including physician notes and labs. The study population included all individuals with an index I/R medication in the database between 1 December 2020 and 31 December 2022. The pre- and post-index period for assessment of clinical characteristics (e.g., prior hospitalizations or antibiotic therapy, hospital readmission, etc.) were 90 days pre- and post-initiation date of I/R. Descriptive statistics were used to examine variables of interest.

**Figure:**
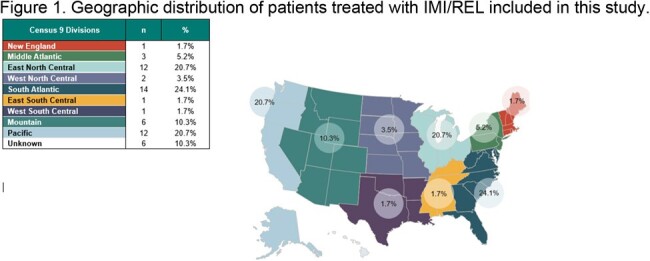

**Results:**

A total of 58 patient charts were reviewed; 41.4% were female and 67.2% were white. The average age at time of admission was 54 years. Mean Charlson Comorbidity Index was 5; 27.6% had a prior hospitalization in the 3 months prior to current infection; 8.6% had presence of bacteremia at time of I/R initiation and 43.1% were diagnosed with sepsis. ICU admission occurred in 55.2% of patients and mean (SD) consecutive days of IMI/REL use was 13 (25) days. Overall, 46.2% of patients had received another novel β-lactam/β-lactamase inhibitor prior to I/R. Most patients (74.1%) did not experience a 30-day hospital readmission after receiving I/R. Among those (25.9%) who did have a readmission, most (66.7%) had a single hospital readmission. The mean length of stay for hospitalization was 39 days. The all-cause in-hospital mortality (ACHM) was 13.8%. 56.9% of the isolates from patients treated with I/R had susceptibility to *Pseudomonas aeruginosa*. Nearly half of the patients (46.6%) had COVID-19 at index.

**Conclusion:**

Among patients who survived to discharge, approximately three-quarters did not experience 30-day hospital readmission. Despite ≥40% non-susceptibility to PsA, ACHM was still low, pointing to the important role of I/R in treatment.

**Disclosures:**

**Emre Yucel, PhD**, Merck: I am a full time Merck Employee and own stocks in the retirement plan provided by Merck.|Merck: Stocks/Bonds (Public Company) **Lulu Lee, PhD**, MERCK: Grant/Research Support **Kushal Modi, MS**, Merck: Grant/Research Support **Yanbing Zhou, PhD**, Merck: I am a full time Merck Employee and own stocks in the retirement plan provided by Merck.|Merck: Stocks/Bonds (Public Company) **Ryan K. Shields, PharmD, MS**, Allergan: Advisor/Consultant|Cidara: Advisor/Consultant|Entasis: Advisor/Consultant|GSK: Advisor/Consultant|Melinta: Advisor/Consultant|Melinta: Grant/Research Support|Menarini: Advisor/Consultant|Merck: Advisor/Consultant|Merck: Grant/Research Support|Pfizer: Advisor/Consultant|Roche: Grant/Research Support|Shionogi: Advisor/Consultant|Shionogi: Grant/Research Support|Utility: Advisor/Consultant|Venatorx: Advisor/Consultant|Venatorx: Grant/Research Support

